# Diagnosis of Agglomeration and Crystallinity of Active Pharmaceutical Ingredients in Over the Counter Headache Medication by Electrospray Laser Desorption Ionization Mass Spectrometry Imaging

**DOI:** 10.3390/molecules26030610

**Published:** 2021-01-25

**Authors:** Mariann Inga Van Meter, Salah M. Khan, Brynne V. Taulbee-Cotton, Nathan H. Dimmitt, Nathan D. Hubbard, Adam M. Green, Gregory K. Webster, Patrick A. McVey

**Affiliations:** 1 Department of Chemistry, Marian University, Indianapolis, IN 46222, USA; mvanmeter409@marian.edu (M.I.V.M.); btaulbeecotton773@marian.edu (B.V.T.-C.); ndimmitt710@marian.edu (N.H.D.); nhubbard324@marian.edu (N.D.H.); 2 College of Osteopathic Medicine, Marian University, Indianapolis, IN 46222, USA; skhan242@marian.edu (S.M.K.); agreen679@marian.edu (A.M.G.); 3 AbbVie Inc., North Chicago, IL 60064, USA; gregory.webster@abbvie.com

**Keywords:** mass spectrometry imaging, MSI, ELDI, agglomeration, crystallinity, pharmaceuticals

## Abstract

Agglomeration of active pharmaceutical ingredients (API) in tablets can lead to decreased bioavailability in some enabling formulations. In a previous study, we determined that crystalline APIs can be detected as agglomeration in tablets formulated with amorphous acetaminophen tablets. Multiple method advancements are presented to better resolve agglomeration caused by crystallinity in standard tablets. In this study, we also evaluate three “budget” over-the-counter headache medications (subsequently labeled as brands A, B, and C) for agglomeration of the three APIs in the formulation: Acetaminophen, aspirin, and caffeine. Electrospray laser desorption ionization mass spectrometry imaging (ELDI-MSI) was used to diagnose agglomeration in the tablets by creating molecular images and observing the spatial distributions of the APIs. Brand A had virtually no agglomeration or clustering of the active ingredients. Brand B had extensive clustering of aspirin and caffeine, but acetaminophen was observed in near equal abundance across the tablet. Brand C also had extensive clustering of aspirin and caffeine, and minor clustering of acetaminophen. These results show that agglomeration with active ingredients in over-the-counter tablets can be simultaneously detected using ELDI-MS imaging.

## 1. Introduction

Quality control of the active pharmaceutical ingredient (API) in tablets is important to maintain optimal bioavailability of the API(s) [1,2,3]. In the original work, mass spectrometry (MS) imaging was used to detect the crystalline form of an active pharmaceutical ingredient (API) in the presence of its amorphous form used in an enabling tablet formulation [4]. Crystallinity can be an issue for BCS Class II/IV drugs if the active drug has a significant difference in kinetic solubility between the crystalline and amorphous forms [5,6]. In pharma, many amorphous forms of poorly soluble drugs go into solution faster than their crystalline forms and thus, this kinetic solubility makes the amorphous form more bioavailable. In these enabling formulations, the presence of crystalline API could be less bioavailable, and its presence makes the tablet less potent.

The distribution of APIs in pharmaceuticals is typically done via spectroscopic methods [7,8,9]. However, selectivity and sensitivity issues associated with these methods exist, especially in formulations with multiple APIs. Mass spectrometry imaging (MSI) utilizes the inherent advantages of mass spectrometry to spatially resolve analytes in solid surfaces. While spectroscopic methods, such as X-ray imaging, are successful in identifying crystals within a tablet, these methods cannot discriminate between ingredients in the formulation [10,11]. The selectivity of mass spectrometry imaging allows for spatially resolved identification of every detected compound in the formulation. As such, unlike with other imaging techniques, Electrospray laser desorption ionization mass spectrometry (ELDI-MS) can readily and selectively profile multiple API ingredients simultaneously within the tablet [12].

MSI of pharmaceuticals has been explored in a limited capacity [13]. Many MSI studies have focused on counterfeits or method development [14,15]. Matrix assisted laser desorption ionization (MALDI) MSI is the most widely used imaging method and has been utilized to image tablets in a limited number of studies [16,17]. One recent study used MALDI-MSI to spatially resolve oxytetracycline tablets [18]. Desorption electrospray ionization (DESI) MSI has also been used to image tablets for proof of concept [19]. However, none of these studies have used MSI to directly diagnose agglomeration in tablets, especially those with multiple APIs. Electrospray laser desorption ionization (ELDI) MSI was previously used by our group to propose levels of crystalline API in standardized acetaminophen tablets [4]. ELDI is similar to MALDI; however, ionization via an applied matrix is replaced by ionization via secondary electrospray post ablation [20]. Agglomeration of acetaminophen was linked to levels of crystalline versus amorphous drug.

The present study uses ELDI-MSI to analyze over the counter (OTC) headache medication to detect agglomeration of multiple APIs in commercially available tablets. Acetaminophen, aspirin, and caffeine are all considered soluble drugs (BCS Class I or I/III). For soluble drugs such as acetaminophen, it likely does not matter if the drug is free, agglomerated, or crystalline. Differences in bioavailability for BCS I/III drugs is typically studied by using dissolution studies, not ELDI-MS or other imaging techniques [5]. The drugs chosen for this investigation are surrogate forms for those expected in enabling formulations. Multiple ELDI-MSI method improvements over the previous work, including increased spatial resolution, are also presented. With these method advancements, individual clusters of active ingredients were better resolved. Selective agglomeration of the APIs in commercially available tablets were readily observed in all samples analyzed of two out of the three brands tested.

## 2. Results

### 2.1. Method Advancements

Multiple method advancements were made to the ELDI-MSI method compared to our previous work. Spatial resolution was improved 100% from 100 µm lateral resolution to 50 µm. This was accomplished through an additional flush focusing optic and alterations to the beam expander. A Thermo LTQ XL mass spectrometer was used for increased sensitivity to accommodate the increase in spatial resolution via a subsequent decrease in ablated material. While spectral resolution was decreased with the use of a linear ion trap mass spectrometer, all tablet formulations are well-established, and identification of analytes via MSMS was done for confirmation of peak assignments.

Previously, a home-built electrospray system was utilized for post-ablation ionization. This home-built system was limited in capabilities as it had no sheath gas flow and required the complete removal of the source enclosure. Without the source enclosure, air currents negatively affected ablation and electrospray reproducibility and sensitivity. The present study uses a modified Thermo electrospray source mounted in the same plane as the MS-inlet within an enclosed Thermo electrospray housing. This allowed for a nitrogen sheath gas flow around the electrospray to better facilitate direction of extracted material into the MS-inlet. The enclosure around the electrospray source eliminated air current issues, which led to enhanced stability of the ELDI signal.

Typically, the thermo electrospray source is mounted through the top of the source enclosure at an angle. We found that sensitivity was severely impacted by this configuration. As the laser strikes the sample surface, it creates a “plume” of ablated material. This plume naturally diffuses as its distance from the sample surface increases. Therefore, ablated particulates must interact with the electrospray droplets before diffusion takes place for efficient extraction into the mass spectrometer. However, if the sample surface is located too close to the electrospray capillary or MS-inlet, then a desorption effect from the electrospray is observed, thus eliminating spatial information acquired via laser ablation. Due to these issues, specific geometries between the electrospray source, MS-inlet, and sample surface are required for maximizing sample extraction and detection. This modified source housing allows for this enhanced sensitivity while maintaining the spatial information acquired via laser ablation.

A Nd:YAG laser was purchased to operate at 266 nm with a shorter pulse width (<2 ns). This change in wavelength allowed ablation of standard tablets without the need for doped titanium (IV) oxide to facilitate ablation. This change made the method truly matrix-free, as no matrix was applied to the surface of the pills or in the formulation. Finally, commercial tablets were successfully ablated due to the change in laser wavelength, while some OTC tablets had ablation issues at 355 nm.

Previous images taken via ELDI-MSI had an issue with horizontal elongation of signal. This was due to multiple issues. First, a lower lateral resolution did not allow for long “streaks” to be spatially resolved. This was fixed with the decrease of the laser spot size to 25 µm. Second, due to the amount of material being ablated from the tablet surface, there were issues with particulates efficiently being extracted into the mass spectrometer via the secondary electrospray. This was similarly fixed with the decrease in spot size, but also with the pneumatically assisted electrospray. A nitrogen gas flow facilitated the washout of particulates clustering outside the mass spectrometer inlet which previously led to streaking.

### 2.2. ELDI-MSI of Acetaminophen Crystals

**Figure 1:**
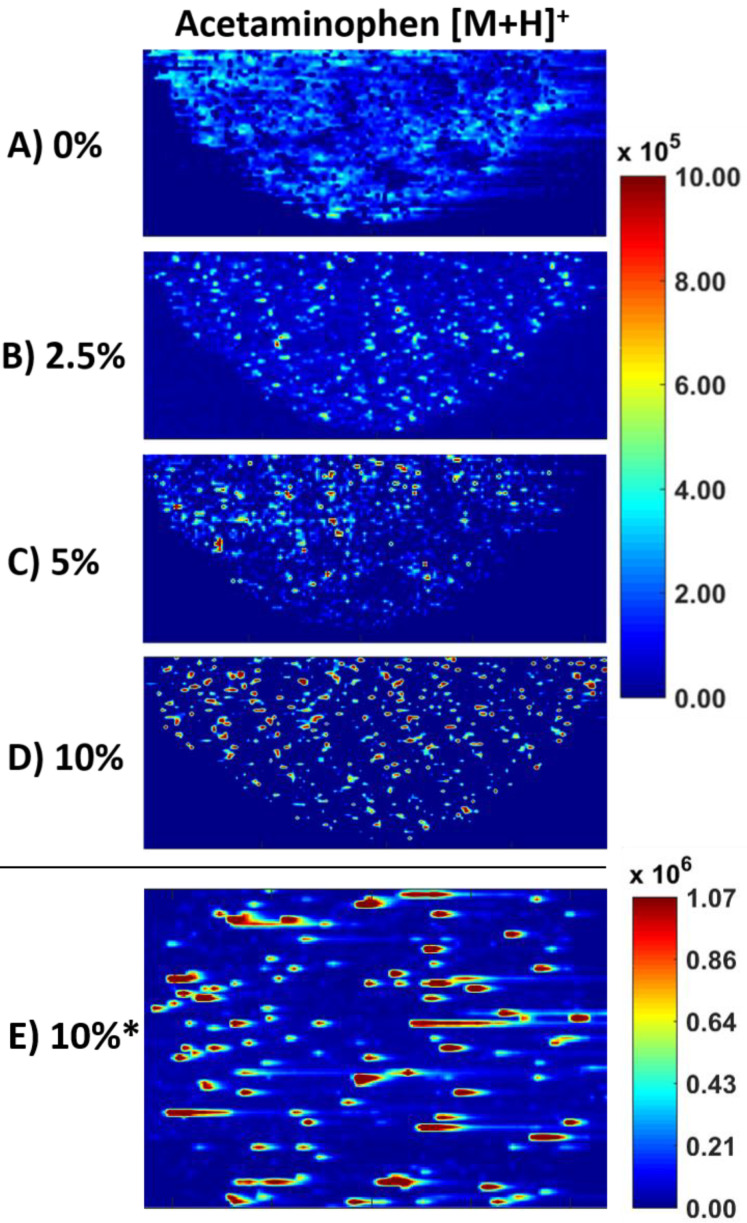
ELDI-MSI (electrospray laser desorption ionization mass spectrometry imaging) of standard acetaminophen tablets at increasing crystallinity percentages. Half of each pill was imaged for 1A-D with the updated method. Acetaminophen was observed at *m/z* 152.07 [M + H]^+^. Images A-D image are set to the same heatmap scaling for ease of analysis. (**A**) Image of 100% amorphous active pharmaceutical ingredient (API) tablet; (**B**) 2.5% crystalline API tablet; (**C**) 5% crystalline API tablet; (**D**) 10% crystalline API tablet. (**E**) Ten percent crystalline API tablet imaged with the previous ELDI-MSI setup. The entire tablet was imaged.

To highlight the method advancements and to better facilitate identification of crystalline material, ELDI-MSI at 266 nm of standard acetaminophen tablets was done similar to our previous work. Acetaminophen tablets with 0, 2.5, 5, and 10% crystalline API were analyzed. Acetaminophen [M + H]^+^ was observed at *m/z* 152.07 in all tablets. Figure 1 shows the distribution of acetaminophen in tablets at the different crystalline percentages set to the same heatmap intensity scaling. Standard tablets with 0% crystallinity, and therefore 100% amorphous API, had a relatively consistent *m/z* 152.07 signal across the pill (Figure 1A). As crystalline API is introduced, agglomeration is observed. At 2.5% crystalline API, higher intensity spots are observed (Figure 1B). At 5% crystalline API, agglomeration becomes more apparent, and the lower intensity signal previously observed across the sample begins to drop off (Figure 1C). At 10% crystalline API, agglomeration is apparent across the entire sample, and a lower intensity API signal is not observed in Figure 1D. There is still a very low API signal (<1 × 10^3^) across all tablet formulations, however, it is not observed in some MS images due to the heatmap scaling used for ease of comparison between them. 

To provide a direct comparison to our previous ELDI-MSI setup, Figure 1E shows a 10% crystalline tablet imaged prior to the method advancements. While agglomeration is observed similar to Figure 1D, streaking of the signal due to high intensity clusters is observed, which makes data analysis difficult. The increase in resolution in Figure 1D allows for individual clusters to be discriminated. Note that Figure 1E is set to a slightly different heatmap, but the difference in maximum intensity is minor. The increase in spatial resolution is apparent between Figure 1D (25 µm lateral) and Figure 1E (50 µm lateral). 

**Figure 2:**
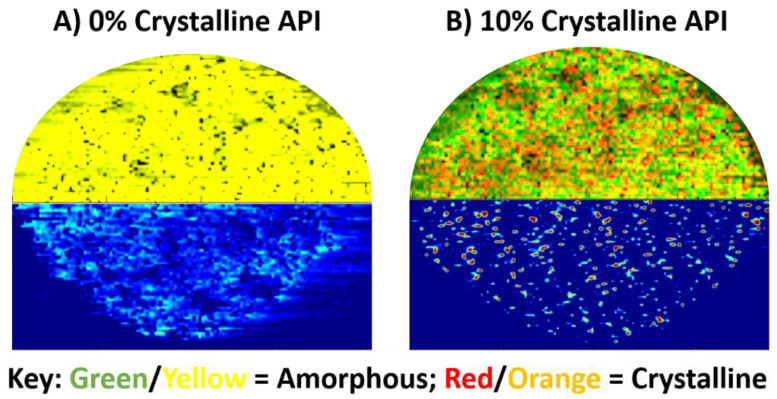
Co-localization plots of acetaminophen and the L90 surfactant (top half) with ELDI-MSI (bottom half) of (**A**) 0% crystalline (100% amorphous) and (**B**) 10% crystalline standard acetaminophen tablets. The co-localization plots on top compare acetaminophen at *m/z* 152.07 and the L90 surfactant at *m/z* 458.42. The MS images on bottom represent the spatial distributions of just the API.

This data is consistent with our previously published results. Streaking issues due to high intensity signals (Figure 1E) are no longer present, allowing for well-resolved clusters to be seen. The higher spatial resolution allows for better discrimination of individual agglomerated spots and potential crystals. The API signal was also compared to the L90 surfactant observed at *m/z* 458.42. The L90 surfactant should only be observed as localized with the amorphous form of the API and not with crystalline API. Figure 2 shows the co-localization (top MS image) of acetaminophen and the surfactant. Yellow or green colorization in the co-localization image represent amorphous acetaminophen. Red or orange colorization in the co-localization image represent crystalline acetaminophen. Figure 2A represents a 100% amorphous tablet, and the API subsequently appears nearly 100% localized with the surfactant as expected. Figure 2B is 90% amorphous, 10% crystalline, and while much of the co-localization image is green or yellow, multiple orange or red spots are observed, representing pixels with signal only from acetaminophen (i.e., crystalline API).

### 2.3. ELDI-MSI of OTC Tablets

Three different brands of over the counter headache medication were analyzed via ELDI-MSI. These are subsequently labeled as brands “A”, “B”, and “C”. Each brand had the same advertised drug loads of the three APIs: Acetaminophen, aspirin, and caffeine. MS images of each API were obtained and compared across the three brands. Brand A was the most expensive of the brands analyzed and had the least amount of agglomeration for all APIs. Brands B and C were similarly priced and advertised to low income households. The following adducts were observed in all three commercial tablets analyzed: Acetaminophen [M + H]^+^ at *m/z* 152.07, aspirin [M − H_2_O + H]^+^ at *m/z* 163.04, and caffeine [M + H]^+^ at *m/z* 195.09.

**Figure 3:**
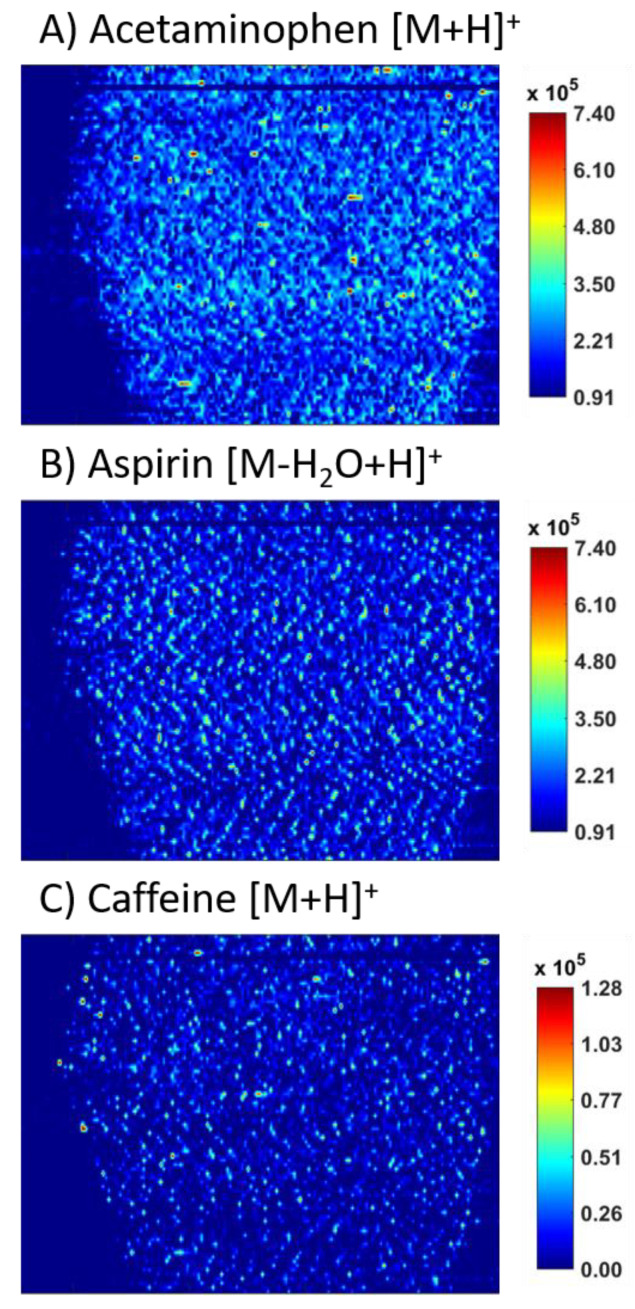
MS images of the three APIs in brand “A” over the counter tablets, including (**A**) acetaminophen, (**B**) aspirin, and (**C**) caffeine.

Figure 3 shows MS images of Brand A tablets. Very little agglomeration was observed for all three APIs with a few higher intensity spots. However, these high intensity spots were only about twice as concentrated as the rest of the API observed across the sample surface. Overall, tablet A had a consistent signal for all APIs across the entire sample surface. Both acetaminophen and aspirin are set to the same heatmap scaling due to both having the same advertised drug load. Caffeine has a lower heatmap scaling to better represent its spatial distributions as it has a much lower drug load and less intensity was observed. Note that on Figure 3 there is a stripe of “empty” pixels located near the top of the MS images. This stripe represents a test of the signal when the laser is turned off and no sample is extracted. Based on the empty signal through those runs, we can be confident that the signal at each peak we associate with the APIs is 100% from our sample.

**Figure 4:**
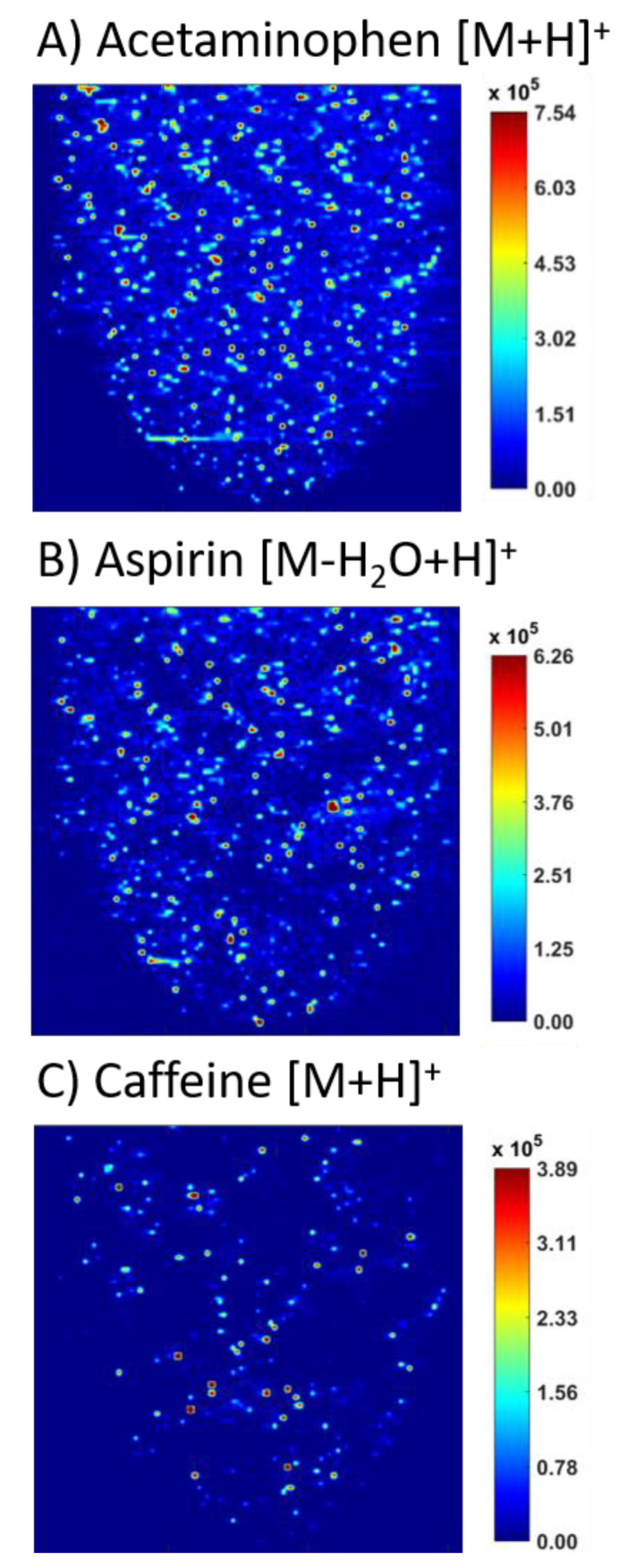
MS images of the three APIs in brand “B” over the counter tablets, including (**A**) acetaminophen, (**B**) aspirin, and (**C**) caffeine.

Figure 4 shows MS images of Brand B tablets. Agglomeration was observed for all APIs at a much higher extent than in Brand A. While signal for acetaminophen is observed throughout the tablet surface (Figure 4A), many high intensity spots are seen. The spatial distribution of aspirin, Figure 4B, shows multiple high intensity spots as well as small areas with minimal signal. Caffeine had the most intense agglomeration of all APIs in tablet B. Nearly all the signal observed for caffeine was located in high intensity spots, with large areas of minimal signal. Areas with high ion intensity for aspirin showed minimal intensity for caffeine. Similarly, high ion intensity pixels of caffeine showed lower ion intensity for aspirin.

**Figure 5:**
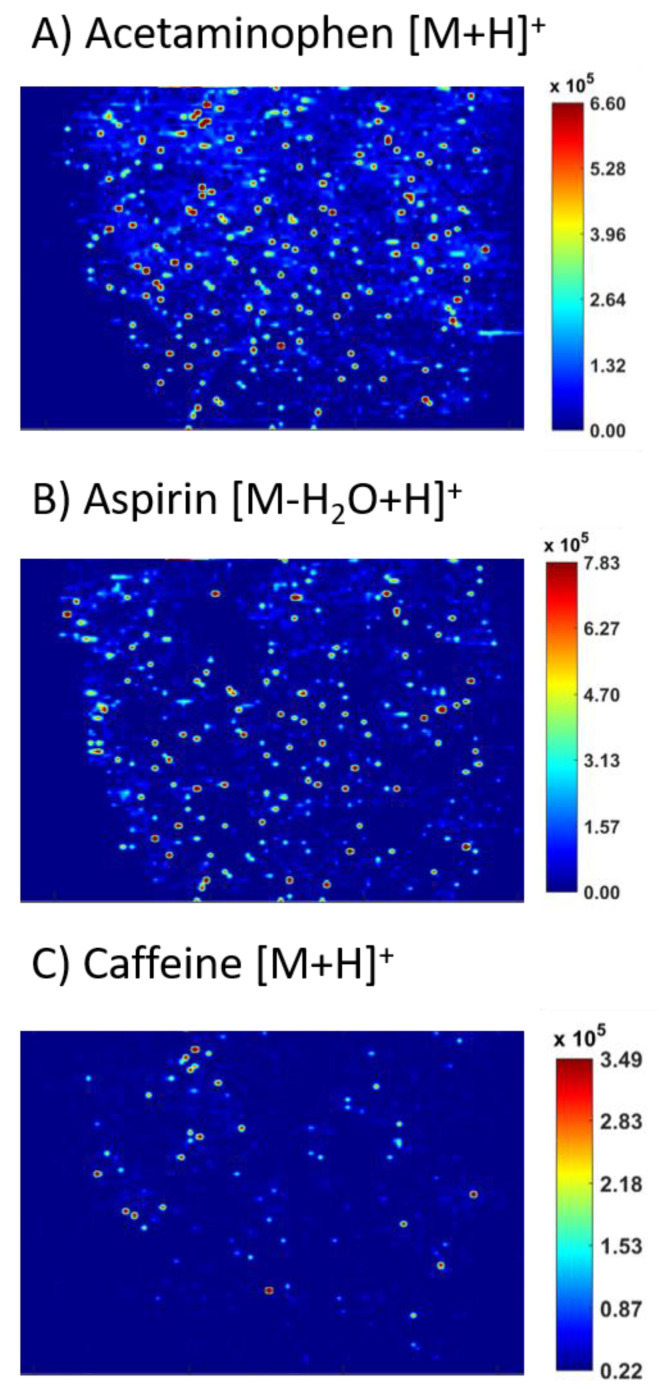
MS images of the three APIs in brand “C” over the counter tablets, including (**A**) acetaminophen, (**B**) aspirin, and (**C**) caffeine.

The third brand tested showed similar results to those seen in Brand B. The agglomeration of all three APIs is apparent for Brand C in Figure 5. Localized areas similar to both distributions were observed in Figure 5A for acetaminophen, as the bottom left area of the image shows more intense agglomeration. Figure 5B shows aspirin with intense agglomeration across the sample. In Figure 5C, caffeine is observed with intense clustering in a few high intensity spots. Aspirin had more intense agglomeration as compared to Brand B tablets with larger areas of minimal signal. Caffeine images for Brand C closely resembled those from Brand B.

## 3. Discussion

This study presents ELDI-MSI method advancements and their use to diagnose agglomeration of multiple APIs within the same tablet. In the original work, we looked for crystallinity to see if we could detect it from amorphous forms. ELDI-MS can be used to detect a change in this state, but not diagnose crystallinity independently. Section 2.2 highlights how the recent advancements to the ELDI-MSI setup allow for better interpretation of crystalline acetaminophen on agglomeration of the API, as seen in Figure 1. Section 2.3 highlights how ELDI-MS can detect drug specific agglomeration even in the presence of other drugs (Figure 3, Figure 4 and Figure 5). Other imaging techniques could not differentiate which drug is agglomerating. In addition, other methods are not sensitive enough to detect crystallinity at this level in a tablet. Thus, for multi active formulations, ELDI-MS is a better choice than present spectroscopic methods, and can be complimentary to those methods for other analyses. This is especially true for formulations with low drug loads, which benefit from the sensitivity offered by mass spectrometry.

The change in laser wavelength, updated optics system, and addition of a nitrogen gas flow around the electrospray allowed for an increase in lateral resolution and a major decrease in previously observed horizontal “streaking” of high intensity signal. A direct comparison of present and previous data is shown in Figure 1. These changes also made the ELDI-MSI method a completely matrix-free, atmospheric pressure method operating at a 25 µm lateral resolution. This is among the best laser ablation spatial resolutions at the ambient pressure available. Without these method advancements, OTC and multi-drug tablets could not be analyzed.

It should be noted that a direct comparison of the agglomeration in the standardized acetaminophen tablets to the OTC headache medication cannot be made. The analysis of the samples in Section 2.2 show agglomeration of the active ingredient with increasing crystallinity. However, the OTC tablets analyzed by ELDI-MSI in Section 2.3 did not have any additional analyses (such as X-ray imaging) to determine crystalline content. The data presented here is to highlight the ability of ELDI-MSI to determine agglomeration of a single API (Section 2.2) as well as discriminate agglomeration of multiple API formulations and in commercially available tablets (Section 2.3). The reason for agglomeration of APIs in the headache medication analyzed would need additional tests to be determined.

The tablet brands purchased were marketed towards low-income households. The generic brand “A” was also found in multiple pharmacies. These tablets, as seen in Figure 3, had minor, if any, agglomeration. This brand was the most expensive purchased ($0.29/pill versus ~$0.04/pill USD) and was the highest quality in terms of even distribution of all three APIs, as observed in the MS images. Brands B and C could not be found in local pharmacies and were only available for purchase online. The advertised APIs were readily observed in both formulations and peak intensities correlated favorably with the listed drug loads and Brand A. However, both brands B and C had extensive agglomeration of all three APIs, especially caffeine. Agglomeration does not indicate decreased performance of these tablets.

These results demonstrate the potential for ELDI-MSI as a quality control device for manufacturing of tablets with multiple APIs. The MS-images allow for spatial discrimination of agglomerated active ingredients that can be compared to spectroscopic results for identification of crystals observed via those methods. These results also model the capabilities of ELDI-MSI for BCS Class II/IV drugs where simple dissolution experiments are not sensitive to low levels of crystallinity that can impact potency [21].

## 4. Materials and Methods

### 4.1. ELDI-MS 

A Thermo Fisher Scientific LTQ XL linear ion trap mass spectrometer was used for data collection. The Thermo electrospray ionization (ESI) source housing was modified to accommodate laser ablation at ambient pressure. The ESI source was mounted through the front window of the source housing with the ESI positioned in the same plane as the mass spectrometer sample inlet. Plastic coverings were mounted around the electrospray and translation stage, and the focusing optics were mounted within the electrospray source housing to fully enclose the ionization source and prevent unwanted air currents.

Data were acquired in the mass range from *m/z* 100 to 2000. Spectra were summed for 0.25 s per image position. The LTQ XL was operated in Xcalibur and LTQ Tune Plus Version 2.7.0.1103 SP1.

### 4.2. ELDI Source

Samples were ablated with a Nd:YAG laser (Q-Spark-A50, Quantum Light Instruments, Ltd., Mokslininku str. 6A, Vilnius, Lithuania). The fourth harmonic was used at 266 nm. The laser was operated at a pulse repetition rate of 10 Hz, with a <2 ns pulse width, and an energy of 800 µJ/pulse (before focusing). The laser beam passed through a fused silica right-angle prism and then was focused onto the sample by a beam expander, followed by two flush plano-convex focusing lenses (UV coated fused-silica, focal length 75 mm) with a nominal spot size of ~25 µm.

Pill samples were mounted on a glass slide using double-sided tape. No matrix was applied. Samples were placed approximately 8 mm below the ESI-sample cone axis on a computer-controlled translation stage (Z825B, ThorLabs, Inc., Newton, NJ, USA). Samples were translated at 0.4 mm/s beneath the static laser beam horizontally across the surface of the tablet. Approximately 5 mm tablet cross-sections were imaged. The distance between the centers of adjacent ablation tracks was 50 µm, providing a lateral resolution of 50 µm. Each mass spectrometry image pixel represents a total ablated volume of ~25 µm by 50 µm by 30 µm deep. The tablets were irradiated normal to the sample surface with the laser beam axis ~2 mm downstream from the ESI capillary. The ESI tip was ~10 mm from the sample inlet.

A solution of 50% methanol with 0.1% formic acid (99.5% purity, Fisher Scientific, Hampton, NH, USA) was pumped through a 53 µm ID polyimide coated capillary as the ESI solution via the attached LTQ XL pump. All data were acquired in positive mode. The ESI voltage was +5.00 kV, drying gas flow rate was 11 L/min, and nebulizer gas pressure was 35 psi. The sample inlet was kept at 100 °C with a N_2_ curtain gas flow of 5 L/h.

### 4.3. Data Handling

Spectra were generated from total ion chromatograms (TIC) combined by the Xcalibur software (V 4.0, Thermo Fisher Scientific Inc., Waltham, MA, USA). The “.raw” data files were converted to mzML files by Proteowizard Mass Converter Tool [22]. The mzML files were then combined into an imzML file using imzMLconverter V1.3 [23]. This combined image file was then viewed, and images were generated from MSiReader V1.01 via the W.M. Keck FTMS Laboratory [24]. All images made within MSiReader had Linear^2^ interpolation for image clarity, and used the “Jet” colormap/false color appearance. Co-localization images were created with MSiReader.

### 4.4. OTC Tablet Information

Three different brands of “headache relief” tablets were purchased via Amazon.com, Inc. Three different bottles were purchased of each brand, with a minimum of 10 tablets analyzed from each bottle for each brand. All brands had the same advertised APIs and drug loads: 250 mg acetaminophen, 250 mg aspirin, and 65 mg caffeine. Brand “A” was the most expensive at $0.29/pill USD. Tablets weighed 0.6716 g on average. The main inactive ingredients were corn starch, crospovidone, and hypromellose. The pills were “tablet” shaped with rounded sides that were shaved down prior to analysis to remove any outer coating. The date of manufacture was 12/18.

Brand “B” cost $0.03/pill USD. Tablets weighed 0.6873 g on average. The main inactive ingredients were colloidal silicon dioxide, hypermellose, and microcrystalline cellulose. The pills were “tablet” shaped with rounded sides that were shaved down prior to analysis to remove any outer coating. The date of manufacture was 04/19.

Brand “C” cost $0.05/pill USD. Tablets weighed 0.6796 g on average. The main inactive ingredients were corn starch, crospovidone, and hypromellose; same as brand “A”. These pills were rounded and thicker than the other brands. The tablets were shaved down to remove any outer coating and obtain the same overall pill thickness as the aforementioned brands. The date of manufacture was 06/19.

### 4.5. Standard Tablet Formulation

Standard acetaminophen tablets (100 mg API) were created by AbbVie at 0, 2.5, 5, and 10% crystalline API. Standard tablets were circular in shape with half of each tablet imaged (5 mm radius). The acetaminophen (Sigma, USP grade) was initially crystalline. The hot melt extrusion (HME) process dissolved the drug into the tablet filler copovidone (Plasdone S-630 polymer, Ashland, Columbus, OH) to become an amorphous solid dispersion (ASD). Lauroglycol (7%) and tween-80 (3%) surfactants were incorporated into the extrudate. The ASD amorphous acetaminophen was then blended with the crystalline acetaminophen to get the various crystalline spiked percentages, with additional copovidone added to act as filler. All blends were extruded on a Thermo Process 11 Hygienic TSE using a gravimetric twin screw feeder. The extrusion process was kept the same for all blends.

### 4.6. Safety Considerations

Appropriate laser safety goggles were worn during ELDI experiments. The modified source housing was kept behind a plastic shield to reduce the risk of shock.

## 5. Conclusions

This is the first study applying MSI on OTC tablets for quality control of agglomerated API within the pharmaceutical industry and beyond the identification of counterfeit drugs. Method improvements over previous studies allowed for greater discrimination of crystalline API in standard tablets. Independent agglomeration and identification of multiple active ingredients was observed in OTC tablets, which has not been accomplished via spectroscopic methods. This illustrates the advantages of ELDI-MSI for concurrent spatial distribution analysis, detection, and identity confirmation of multiple dosage formulations.

This study also models the applicability of ELDI-MSI to study crystalline BCS class II/IV compounds and to detect low levels of crystallinity. MSI is shown to be an advantageous method, which utilizes the selectivity and sensitivity of mass spectrometry to compliment current methods of low-level crystalline API detection in amorphous dosage formulations. The issue of crystallinity in enabling formulations is of significant interest for manufacturing and stability testing of formulations.

Recently, X-ray microscopy has been shown to quantitate crystalline API in tablets [25]. X-ray and MSI are complimentary techniques, as the former can determine crystalline structure, but is not a selective technique and therefore cannot determine what substance make up observed crystals. Future and on-going studies include the comparison of ELDI-MSI with X-ray imaging of the same tablets to identify crystals; further method advancements for increased spatial resolution; and quantification of crystalline API through MSI and X-ray collaboration.

## Data Availability

All acquired mass spectrometry images and data available upon written request to the corresponding author.

## Sample Availability

Samples of the compounds are not available from the authors.
